# Development of a Competitive ELISA for Detecting Antibodies Against Pseudorabies Virus Glycoprotein D

**DOI:** 10.1155/tbed/1263531

**Published:** 2025-11-13

**Authors:** Lei Na, Yue Sun, Wen-Ying Qiu, Yang Zheng, Wei Ding, Jian-Bo Yang, Yanhe Zhang, Jinqiu Zhang, Yan-Dong Tang

**Affiliations:** ^1^College of Animal Husbandry and Veterinary Medicine, Jiangsu Vocational College of Agriculture and Forestry, Jurong, China; ^2^State Key Laboratory for Animal Disease Control and Prevention, Harbin Veterinary Research Institute of Chinese Academy of Agricultural Sciences, Harbin, China; ^3^Institute of Veterinary Immunology and Engineering, Jiangsu Academy of Agricultural Sciences, Nanjing, China; ^4^College of Veterinary Medicine, Sichuan Agricultural University, Chengdu, China

**Keywords:** competitive ELISA (cELISA), glycoprotein D (gD), monoclonal antibody, pseudorabies virus (PRV), serological diagnosis

## Abstract

Pseudorabies virus (PRV) causes substantial economic losses in the global swine industry. Serological diagnosis plays a crucial role in its eradication. Here, we developed a competitive enzyme-linked immunosorbent assay (cELISA) to detect antibodies against PRV glycoprotein D (gD). First, the recombinant gD ectodomain was expressed and purified to immunize mice, resulting in the generation of a monoclonal antibody (mAb 1D11) that targets gD. Subsequently, this antibody was conjugated with horseradish peroxidase (HRP), serving as the competing reagent. The cELISA was optimized under ideal conditions. Furthermore, validation using 204 swine serum samples—comprising 110 PRV-positive and 94 PRV-negative samples—demonstrated a high sensitivity and specificity, with a cutoff value of 46.16% inhibition determined by receiver operating characteristic (ROC) analysis (area under the curve = 0.995). Importantly, no cross-reactivity was observed with antibodies against other tested swine viruses. Both intra- and inter-assay coefficients of variation were found to be less than 10%, confirming high reproducibility of the assay results. When compared to a commercial PRV glycoprotein B (gB) ELISA kit (IDEXX), our cELISA exhibited strong agreement with *κ* = 0.90. This robust, specific, and sensitive cELISA provides a reliable tool for large-scale monitoring of PRV antibodies.

## 1. Introduction

Pseudorabies, caused by pseudorabies virus (PRV), is a highly contagious and economically devastating disease in the swine industry, leading to high mortality in piglets and reproductive failure in sows [1]. Pigs serve as the primary natural host for PRV. Infected pigs display a range of clinical manifestations upon PRV infection, which can include respiratory distress, neurological dysfunction, reproductive failure, and mortality [2]. The severity of these symptoms is influenced by factors such as age, immunological competence, and viral genotype [2, 3]. In recent years, increasing evidences have demonstrated that PRV possesses the ability for cross-species transmission, infecting ruminants, carnivores, and rodents with near-uniform lethality [4–6]. More concerningly, outbreaks of genotype II variants have heightened spillover risks, including sporadic human infections. This situation underscores the urgent need for the eradication of PRV [7, 8]. The achievement of PRV purification primarily involves the application of vaccines and serological testing. Vaccination serves as a cornerstone in controlling animal diseases; however, to effectively eliminate this virus, serological diagnostics are also crucial [9, 10]. Therefore, it is imperative to develop a comprehensive approach to combat PRV through serological diagnosis, which will facilitate the effective eradication of this virus.

Currently, several serological assays are available for detecting PRV-specific antibodies, including virus neutralization tests (VNTs), indirect enzyme-linked immunosorbent assay (ELISA), and competitive ELISA targeting glycoproteins such as glycoprotein B (gB) and gE. While VNT remains the gold standard for its high specificity, it is labor-intensive and unsuitable for high-throughput screening. Indirect ELISAs offer higher throughput but may be affected by cross-reactivity and variability in polyclonal antisera. Commercial gB–based ELISAs are widely used for general antibody detection, whereas gE-based assays are employed in Differentiating Infected from Vaccinated Animals (DIVA) strategies. However, there is a growing need for assays targeting other immunodominant and conserved antigens, such as glycoprotein D (gD), which plays a critical role in viral entry and elicits strong neutralizing antibody responses. Importantly, gD is highly conserved among diverse PRV genotypes, including emerging variants, making it a robust candidate for broad-spectrum serological detection. While gB is also immunogenic and commonly targeted in commercial assays, gD offers a complementary approach that may capture a distinct subset of the humoral response, particularly in animals with differential antibody profiles due to infection or vaccination history. Thus, a gD–based competitive ELISA (cELISA) not only expands the diagnostic toolkit but also provides a universal target for monitoring PRV exposure across genetically heterogeneous strains. This study aims to develop a gD–based cELISA to complement existing tools and enhance PRV serological surveillance.

To this end, here, we have prepared a mouse monoclonal antibody (mAb) against the gD protein of PRV and labeled it with horseradish peroxidase (HRP) for use in the cELISA assay. This approach offers several advantages, including simplicity, speed, and high sensitivity. This method is expected to provide a rapid and reliable tool for the detection of PRV gD antibodies, contributing to the improvement of diagnostic strategies for the control and prevention of swine pseudorabies disease.

## 2. Materials and Methods

### 2.1. Cells, Viruses, Plasmids, and Serum

HEK293T cells, HEK293F cells, SP2/0 myeloma cells, and Vero E6 cells were maintained in our laboratory. PRV strains HeN1 (GenBank Accession Number KP098534), SC (GenBank Accession Number KT809429), TJ (GenBank Accession Number KJ789182), and Bartha K61 (GenBank Accession Number JF797217) were as described in previous works [11–16]. Plasmid pB513B was as described in previous works [16, 17]. Additionally, serum samples against other common porcine viruses, such as porcine reproductive and respiratory syndrome virus (PRRSV), classical swine fever virus (CSFV), porcine circovirus type 2 (PCV2), porcine epidemic diarrhea virus (PEDV), porcine parvovirus (PPV), and African swine fever virus (ASFV), were used to evaluate the specificity of the cELISA.

### 2.2. Expression and Purification of gD Protein

The extracellular domain (amino acids 1–355) of the gD protein from the PRV HeN1 strain was expressed using the HEK293F suspension cell line. The codon-optimized gD gene was cloned into the pB513B vector. Subsequently, it was co-transfected with the PB transposase helper plasmid (System Biosciences, USA) into adherent HEK293F cells [17]. Forty-eight hours post-transfection, the medium was replaced with fresh medium containing 1 µg/mL puromycin (Gibco, USA), with changes every 2 days. Then the engineered cells were adapted to suspension culture. After 5 days of suspension culture, the supernatant was harvested. Purification was performed using Ni-NTA affinity chromatography resin (GenScript, USA) according to the manufacturer's instructions. The purified protein was verified by SDS-PAGE and Western blotting.

### 2.3. Western Blotting

Western blotting was performed as previously described [18, 19]. In brief, purified PRV gD protein or cell lysates were subjected to SDS-PAGE, and proteins were transferred onto a PVDF membrane (Millipore, USA). The membrane was blocked with 5% nonfat milk in PBS at room temperature for 1 h. After washing three times with PBS containing 0.05% Tween-20 (PBST), it was incubated overnight at 4°C with mAb supernatant or mouse anti-Flag antibody. Following three washes with PBST, the membrane was incubated with DyLight 800-conjugated goat anti-mouse IgG antibody (SeraCare, USA) at room temperature for 1 h. Protein bands were finally detected using the Odyssey CLX imaging system.

### 2.4. Animal Immunization

BALB/c mice were immunized subcutaneously with purified PRV gD protein (100 µg per mouse) three times at 14-day intervals. The primary immunization was emulsified in Freund's complete adjuvant (Sigma, USA), while the secondary and tertiary immunizations used Freund's incomplete adjuvant (Sigma, USA). 1 week after the final immunization, blood was collected from the tail vein, serum was separated, and antibody titers were determined by indirect ELISA. In brief, ELISA plates were coated with PRV gD protein (100 ng/well) overnight at 4°C. After washing three times with PBST, plates were blocked with PBS containing 5% nonfat milk (200 µL/well) at 37°C for 2 h. Following three PBST washes, serially diluted mouse serum (1:500 to 1:32,000; 100 µL/well) was added and incubated at 37°C for 1 h. Subsequently, HRP-conjugated goat anti-mouse IgG secondary antibody (1:10,000 dilution; Zhongshan Golden Bridge, China) was added (100 µL/well) and incubated at 37°C for 1 h. After three final PBST washes, 100 µL of 3,3′,5,5′-tetramethylbenzidine (TMB) substrate solution was added per well and incubated at 37°C for 15 min. The reaction was stopped with 50 µL of 0.5 M HCl per well, and the OD_450_ value was measured to determine serum titers.

### 2.5. Preparation of Monoclonal Antibodies

Mice exhibiting high serum titers were selected for a final intravenous booster immunization without adjuvant, administered 3 days prior to cell fusion. Briefly, immunized mice were euthanized, and splenocytes were aseptically harvested. Splenocytes were then fused with SP2/0 myeloma cells using polyethylene glycol 1450 (PEG 1450; Sigma-Aldrich, USA). The fused cells were cultured in DMEM supplemented with 20% fetal bovine serum (FBS) and 1% hypoxanthine–aminopterin–thymidine (HAT; Sigma–Aldrich, USA). Following cell fusion, the resulting hybridomas were cultured in 96-well plates. A total of 576 hybridoma clones were initially screened for reactivity against PRV gD using indirect ELISA as described in Section 2.4. Clones exhibiting an S/N ratio (OD_450_ of sample well/OD_450_ of SP2/0 supernatant control well) greater than 2.1 were considered positive. From the initial screening, 12 positive clones were identified. These positive clones were further characterized by immunofluorescence assay (IFA) and western blotting to confirm specificity for gD. The 1D11 clone, which demonstrated strong and consistent reactivity in both assays, was selected for further development. To ensure monoclonality and stability, the 1D11 clone was subcloned three times by limiting dilution. The reactivity of mAb supernatants was characterized by IFA and western blotting. Furthermore, the reactivity of mAb supernatants with other PRV strains (TJ, SC, and Bartha K61) was assessed by IFA.

### 2.6. Plasmid Transfection and Virus Infection

HEK 293T cells were seeded in 12-well plates. Upon reaching 70%–80% confluency, cells were transfected with gD plasmids using JetPRIME Transfection Reagent (Polyplus, France) according to the manufacturer's instructions. Fresh medium was replaced 4–6 h posttransfection, and cells were harvested for IFA or Western blotting 24 h later. Vero E6 cells were seeded in 12-well or 96-well plates. Upon reaching 90%–100% confluency, cells were infected with indicated PRV strains at a multiplicity of infection (MOI) of 0.01. When significant cytopathic effect (CPE) was observed, cells were fixed for IFA or lysed for western blotting.

### 2.7. IFA

HEK293T cells (24 h post-transfection) or Vero E6 cells (upon significant CPE postinfection) were washed three times with PBS and fixed with 4% paraformaldehyde at room temperature for 15 min, as described previously [15, 17, 20–22]. After three PBS washes, cells were permeabilized with 0.25% Triton X-100 (Beyotime, China) for 10 min at 4°C and blocked with 2% bovine serum albumin (BSA) at 37°C for 1 h. Following three PBS washes, cells were incubated with mAb supernatant, SP2/0 supernatant (negative control), or mouse anti-Flag antibody (Proteintech, USA) at 37°C for 1 h. After three PBS washes, cells were incubated with fluorescein isothiocyanate (FITC)-conjugated goat anti-mouse IgG secondary antibody (1:200 dilution; Sigma, USA) at 37°C for 1 h. Cells were washed three times with PBS, counterstained with 4′,6-diamidino-2-phenylindole (DAPI) to visualize nuclei, and fluorescence signals were observed using an inverted fluorescence microscope.

### 2.8. Antibody Purification and Labeling

Ten-week-old BALB/c mice were primed via intraperitoneal injection of 300 µL liquid paraffin. 1 week later, hybridoma cells that stably secrete gD-specific antibodies were injected intraperitoneally at a concentration of 1 × 10^6^ cells per mouse. Ascitic fluid was harvested 7 days post-injection. The collected ascites underwent centrifugation at 10,000 × *g* for 10 min at 4°C. The supernatant was then collected and purified using Protein A/G magnetic beads (GenScript, USA) in accordance with the manufacturer's protocol. The efficiency of purification was evaluated by SDS-PAGE.

Purified mAb from ascites was conjugated to HRP using the periodate oxidation method [23]. In brief, HRP powder (Sigma, USA) was dissolved in ddH_2_O to a concentration of 1 mg/mL. 1 ml of HRP solution was mixed with 100 µL of freshly prepared sodium periodate (NaIO_4_, 60 mM; Sigma, USA) and incubated at 4°C for 30 min. Subsequently, 100 µL of ethylene glycol (160 mM) was added and incubated at room temperature for 30 min in the dark to terminate the reaction. 1 mg of purified mAb was added, mixed thoroughly, and the mixture was transferred to a dialysis bag (molecular weight cutoff: 8,000–14,000 Da). Dialysis was performed against carbonate-bicarbonate buffer (50 mM, pH 9.5) at 4°C for 14 h. After dialysis, 40 µL of sodium borohydride (NaBH_4_, 5 mg/mL) was added to the mixture and incubated at 4°C for 2 h. An equal volume of chilled saturated ammonium sulfate solution (pH 7.2) was added slowly with stirring at 4°C for 30 min. The precipitate was collected by centrifugation at 10,000 × *g* for 10 min, dissolved in PBS (pH 7.4), and dialyzed against PBS at 4°C for 14 h. After dialysis, the solution was centrifuged at 10,000 × *g* for 5 min, and the clarified supernatant containing the HRP-conjugated mAb was collected. The labeling efficiency was verified by direct ELISA using gD protein-coated plates.

### 2.9. Development of the cELISA

Checkerboard titration was employed to ascertain the optimal concentrations of the coating antigen and the HRP-conjugated mAb for the cELISA. The purified PRV gD protein was diluted in carbonate-bicarbonate buffer (pH 9.6) to achieve concentrations ranging from 4 µg/mL to 0.25 µg/mL (specifically, 4, 3, 2, 1, 0.5, and 0.25 µg/mL) and subsequently coated onto a 96-well ELISA plate at a volume of 100 µL per well overnight at 4°C. Following washing with PBST, the plates were blocked with a solution of nonfat milk at a concentration of 2% (200 µL/well) at 37°C for 1.5 h. PRV-positive standard serum (designated as P), sourced from pigs infected with the HeN1 strain, along with PRV-negative standard serum (denoted as N), obtained from specific pathogen-free (SPF) pigs maintained in our laboratory, were diluted in a ratio of 1:2. Equal volumes of either diluted positive or negative standard serum were mixed with serially diluted HRP-conjugated mAb at dilutions of 1:50, 1:100, 1:200, 1:400, and finally at a dilution of 1:800 before being added to each well at a volume of 100 µL per well. The plates were then incubated at 37°C for 30 min. After three washes using PBST, TMB substrate solution (Solarbio, China) was introduced into each well at an amount of 100 µL/well and allowed to incubate in darkness at 37°C for 15 min. The reaction was terminated by adding 50 µL/well of 0.5 M HCl, and optical density readings were taken at OD_450_. The N/P ratio was calculated by dividing the OD_450_ nm value of the negative serum by that of the positive serum (N/P = ODneg/ODpos). The combination yielding the highest N/P ratio was designated as representing the optimal coating concentration of antigen and the ideal working dilution of HRP-mAb. Subsequent optimization steps were performed using the optimal antigen concentration and HRP-mAb dilution. Parameters optimized included antigen coating time (37°C for 2 h vs. 4°C for 12 h), blocking solution concentration (5%, 2%, or 1% nonfat milk), blocking time (2, 1.5, or 1 h), test serum dilution (1:2, 1:4, 1:8, and 1:16), incubation time of the test serum/HRP–mAb mixture (15, 30, and 45 min), and substrate development time (5, 10, and 15 min). To assess the inhibition of test serum samples relative to the negative control, the percentage inhibition (PI) was calculated using the following formula: PI(%) = [(ODneg − ODtest)/ODneg] × 100. Where ODneg is the mean OD_450_ nm of negative control serum and ODtest is the OD_450_ nm of the test serum.

### 2.10. Determination of Cutoff Value, Specificity, and Sensitivity of the cELISA

The optimized cELISA was used to analyze 110 PRV-positive and 94 PRV-negative clinical swine serum samples. OD_450_ nm values were measured, and PI values calculated. Receiver operating characteristic (ROC) curve analysis and interactive dot diagram analysis were conducted using MedCalc software (v20.2) to determine the optimal cutoff value for maximum sensitivity and specificity. The method's specificity was evaluated by testing serum samples positive for six other common swine pathogens (PRRSV, CSFV, PCV2, PEDV, PPV, ASFV) with the optimized cELISA protocol. Sensitivity was assessed through twofold serial dilutions (1:2 to 1:512) of three PRV-positive serum samples (#038, #073, #44) and one negative sample (#066). Samples #038 and #073 came from pigs challenged with PRV HeN1; sample #44 was from a pig challenged with PRV SC.

### 2.11. Reproducibility of the cELISA

Three replicates of a strong positive, a medium positive, a weak positive, and a negative serum sample were evaluated on the same ELISA plate (intra-assay, *n* = 3 wells per sample) as well as on three independently coated ELISA plates (interassay, *n* = 3 plates per sample) utilizing the optimized cELISA protocol. The coefficient of variation (CV) was calculated for each group of samples.

### 2.12. Comparison With a Commercial Kit

A total of 115 clinical swine serum samples were analyzed using the established cELISA and a commercially available PRV gB antibody detection kit from IDEXX Laboratories, USA. The results obtained from both assays were compared to evaluate the level of concordance between them.

### 2.13. Ethics Statement

SPF female BALB/c mice (6–8 weeks old) were purchased from Liaoning Changsheng Biotechnology Co., Ltd. All animal experiments were conducted in Animal Biosafety Level 2 (ABSL-2) facilities at the Nanjing Agricultural University, in compliance with institutional animal care and use guidelines. The study protocol was reviewed and approved by the Ethics Committee of Nanjing Agricultural University (Approval ID: NJAULLSC2024012).

### 2.14. Statistical Analysis

Data were analyzed and visualized utilizing GraphPad Prism (version 8.0) and MedCalc (v20.2). Cohen's Kappa coefficient was employed to assess the agreement between the results obtained from cELISA and those from the commercial kit. Coefficients of variation (CVs) were calculated to evaluate both intra-assay and inter-assay reproducibility. Statistical comparisons were conducted using Student's *t*-test in GraphPad Prism 8.0; significance levels were indicated as *⁣*^*∗*^*p* < 0.05, *⁣*^*∗∗*^*p* < 0.01, *⁣*^*∗∗∗*^*p* < 0.001, *⁣*^*∗∗∗∗*^*p* < 0.0001, and ns (not significant).

## 3. Results

### 3.1. Expression and Purification of Recombinant PRV gD Protein

**Figure 1:**
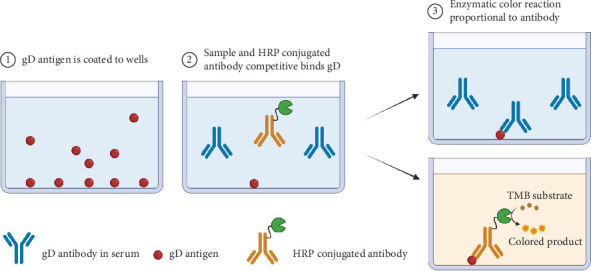
Schematic representation of the competitive enzyme-linked immunosorbent assay (cELISA) method. The schematic illustrates the principle of cELISA: antigen coating, blocking, simultaneous addition of test serum and enzyme-labeled monoclonal antibody (mAb), competition for binding sites, and detection. Created in BioRender. Li, L. (2025) https://BioRender.com/ozel05q.

**Figure 2:**
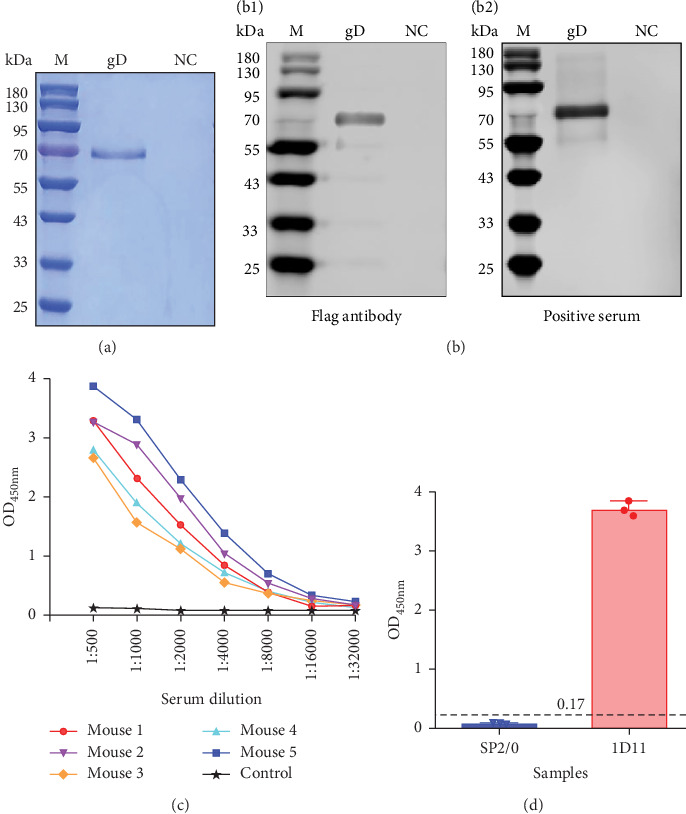
Purification and characterization of PRV gD protein and production of monoclonal antibodies. (a) SDS-PAGE analysis of purified gD protein. gD protein purified via Ni-NTA affinity chromatography from the supernatant of HEK 293F cells stably expressing pB513B-gD; NC: supernatant from untransfected HEK 293F cells. (b) Western blot analysis of purified gD protein. (B1) Probing with mouse anti-Flag primary antibody (1:5000 dilution). (B2) Probing with serum from a gD-immunized mouse (1:2000 dilution). (c) Serum antibody titers of mice following three immunizations with gD protein, determined by direct ELISA. Mouse 1–5: mice immunized with gD protein; control: negative control mouse. (d) Screening of hybridoma clones stably secreting gD-specific antibodies by direct ELISA (representative clone 1D11 shown). The S/N ratio was calculated as the OD_450_ value of the sample well (S) divided by the OD_450_ nm of the SP2/0 supernatant control well (N). Samples with S/N > 2.1 were considered positive.

The cELISA is depicted in Figure 1, which comprises several key steps: antigen coating, blocking, simultaneous addition of test serum and enzyme-labeled mAb, competition for binding sites, and detection. To establish an optimal cELISA method for the detection of gD antibodies, our initial objective was to produce the gD antigen. The codon-optimized extracellular domain of PRV gD was successfully cloned into the PiggyBac transposon system with purification Tag [16, 17, 21, 22]. Next, we established a stable cell line for the expression of gD and successfully produced it as a secreted His-tagged fusion protein utilizing the HEK293F suspension culture system. This mammalian expression system was selected due to its considerable advantages in enabling the rapid production of properly folded proteins with appropriate post-translational modifications—an essential requirement for viral glycoproteins. The cell culture supernatant containing the secreted gD protein was collected and subsequently purified using Ni-NTA affinity chromatography (Figure 2A). The identity of the purified protein was verified through Western blot analysis employing an anti-Flag mAb (Figure 2B, left). Furthermore, we validated this using serum from mice positive for PRV gD. The results further confirm the successful expression of PRV gD (Figure 2B, right). Next, five mice were immunized with the specified antigen; all immunized mice demonstrated a robust antibody response (Figure 2C). Following the hybridoma cell fusion procedures, we successfully identified a hybridoma cell line designated as 1D11, which secretes an antibody exhibiting strong reactivity against the gD antigen (Figure 2D).

### 3.2. 1D11 Monoclonal Antibody Reacted With Viral gD

**Figure 3:**
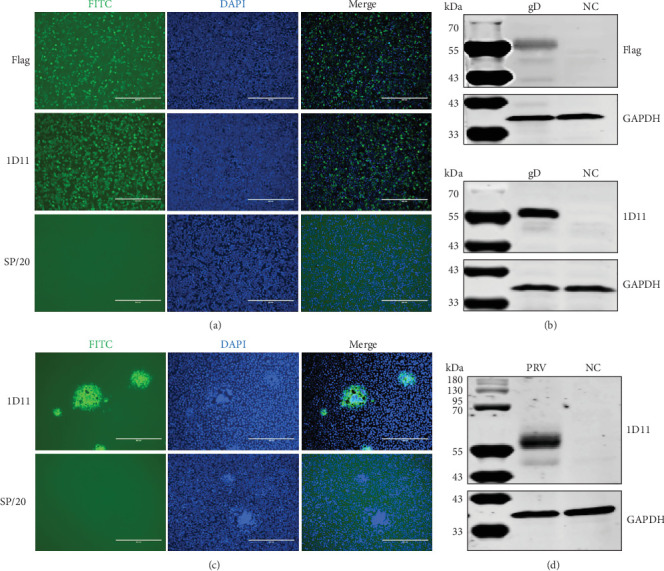
Characterization of monoclonal antibody reactivity. HEK 293T cells were transfected with plasmid pB513B-gD encoding the PRV gD ectodomain (aa 1–355). Reactivity of 1D11 hybridoma supernatant with gD protein was assessed by immunofluorescence assay (IFA) (a) and western blotting (b). Mouse anti-Flag antibody served as a positive control. SP2/0 supernatant served as a negative control. Mouse anti-GAPDH antibody served as a loading control. Vero E6 cells were infected with PRV HeN1 strain at an MOI of 0.01. Reactivity of 1D11 hybridoma supernatant with virus-infected cells was assessed by IFA (c) and WB (d). SP2/0 supernatant served as a negative control. Mouse anti-GAPDH antibody served as a loading control.

Next, we evaluated the specificity of the 1D11 antibody in its reaction with gD. To this end, a gD expression plasmid was transfected into HEK293T cells [24–26]. The reactivity of 1D11 with the gD protein was assessed using an IFA, with anti-Flag antibody serving as a positive control. The results indicated that 1D11 successfully reacted with the gD protein (Figure 3A). We further validated its reaction through Western blotting, utilizing an anti-Flag antibody as a positive control and GAPDH as a loading control. The results indicated that 1D11 exhibited strong reactivity with gD, suggesting that this antibody recognizes a linear epitope (Figure 3B). Next, we assessed whether 1D11 recognizes the virus expressing gD. Vero E6 cells were infected with the PRV HeN1 strain at a MOI of 0.01, using SP/20 supernatant as a control. The results indicated that 1D11 specifically reacted with virus-infected cells (Figure 3C,D).

### 3.3. 1D11 Exhibits Broad Cross-Reactivity With Distinct PRV Genotypes

**Figure 4:**
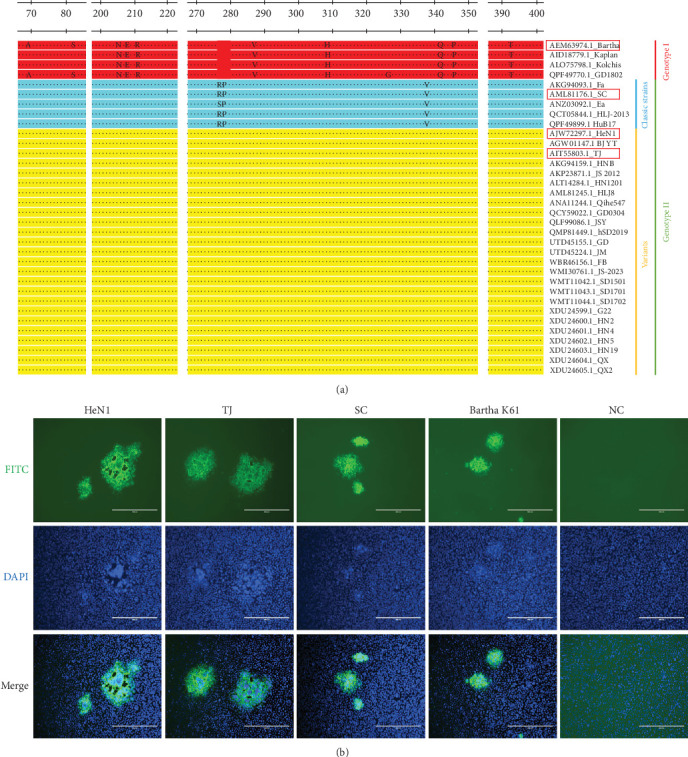
Broad reactivity of the monoclonal antibody with different PRV strains. (a) Amino acid sequence alignment of the gD protein from 34 different PRV strains analyzed using MegAlign software, demonstrating high conservation within 25 variant strains. (b) Vero E6 cells were infected with PRV strains HeN1, TJ, SC, or Bartha K61 at an MOI of 0.01. Reactivity of 1D11 with the different PRV strains was assessed by IFA.

If the antibody used in the cELISA is effective, it should recognize all genotypes of PRV. In fact, PRV is classified into two genotypes: genotype I and genotype II [6]. Furthermore, within the genotype II group, it can be further subdivided into classical strains and variant strains [6]. Therefore, we conducted an analysis of the conservation of gD protein and gD amino acid sequences among different PRV isolates. Our comparison revealed that gD exhibits a high degree of conservation, with only a few mutations observed between distinct genotypes (Figure 4A). Next, in genotype II, we selected a classical SC strain along with two variant strains (HeN1 and TJ strain) [4, 27]. In genotype I, we chose the Bartha K61 strain. Through IFA, we observed that 1D11 reacted with all these strains (Figure 4B). This finding indicates that this antibody recognizes a conserved epitope in PRV.

### 3.4. Establishment of and Optimization of the gD-cELISA

**Figure 5:**
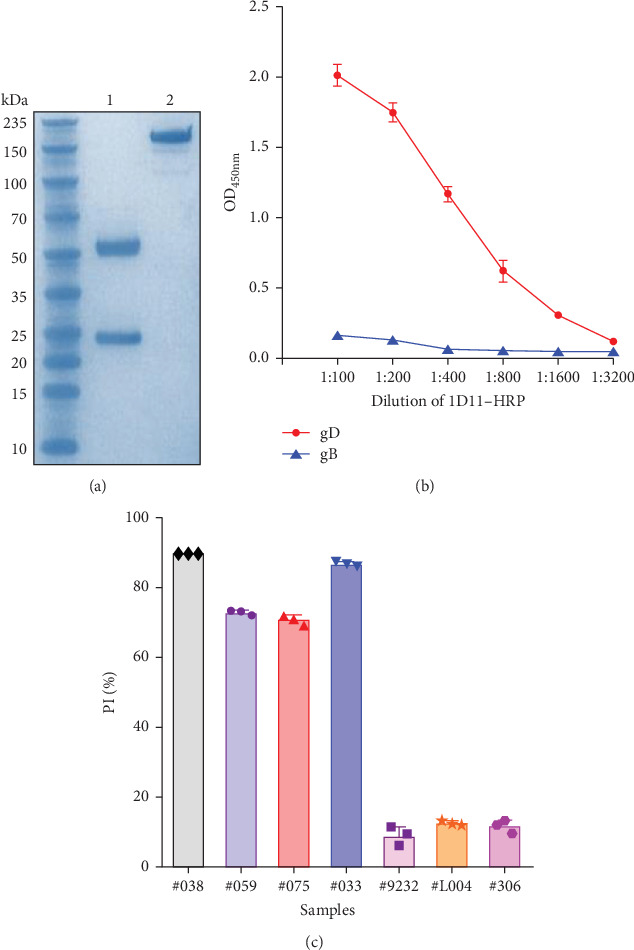
Purification of the monoclonal antibody and assessment of its competitive activity. (a) Purification of mAb 1D11 using Protein A/G magnetic beads. M, protein molecular weight marker; R1 and R2, purified mAb under denaturing and nondenaturing electrophoresis conditions, respectively. (b) Evaluation of HRP conjugation efficiency to mAb 1D11 by direct ELISA. PRV gB protein-coated wells served as a negative control. (c) Assessment of the competitive activity of mAb 1D11-HRP using cELISA. Four PRV-positive swine serum samples (#038, #059, #075, and #033) and three PRV-negative swine serum samples (#9232, #L004, and #306) were tested.

The 1D11 hybridoma cells were transplanted into BALB/c mice for antibody production. Subsequently, the antibodies were purified using Protein A/G magnetic beads. The purification efficiency was evaluated by SDS-PAGE, which indicated that under denaturing conditions, the antibodies displayed standard heavy and light chains. In contrast, under non-denaturing conditions, the antibodies appeared as a larger complex with an increased molecular weight (Figure 5A). Next, the purified 1D11 mAb was conjugated to HRP using the periodate oxidation method. Following HRP labeling, we assessed the HRP conjugation efficiency of mAb 1D11 through direct ELISA. Wells coated with PRV gB protein served as a negative control. The results indicated that mAb 1D11 was successfully labeled (Figure 5B). We then evaluated the competitive activity of mAb 1D11-HRP using cELISA. Four PRV-positive swine serum samples (#038, #059, #075, #033) and three PRV-negative swine serum samples (#9232, #L004, #306) were tested. The results demonstrated that 1D11-HRP exhibits competitive activity (Figure 5C). Consequently, 1D11-HRP may be suitable as a competitive agent for establishing cELISA.

To develop an effective cELISA, varying concentrations of coated gD proteins and HRP-labeled antibodies were added to 96-well plates for the reaction. The optimal working concentrations of the coating antigen (gD) and HRP-labeled antibodies were determined using a checkerboard titration method. The ideal reaction conditions for cELISA utilizing 1D11-HRP antibodies are summarized in Table 1. A concentration of 200 ng/well of purified gD protein was identified as optimal for coating the ELISA plates. The HRP-labeled antibodies were optimally diluted at a ratio of 1:200, resulting in an OD_450_ value of approximately 1.5 in the absence of competing serum antibodies. Additionally, various reaction conditions, including incubation times and temperatures, were optimized to maximize the dynamic range of the assay (Table 1).

### 3.5. Refinement of the Cutoff Value for cELISA

**Figure 6:**
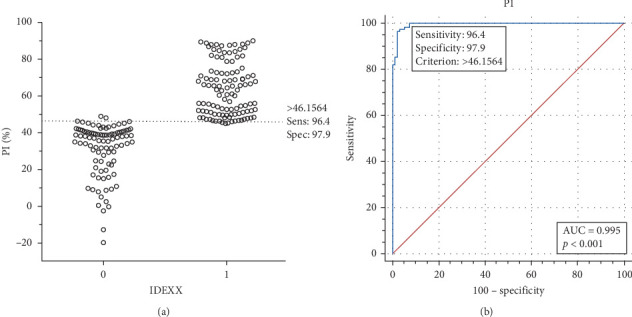
Receiver operating characteristic (ROC) analysis for determining the cut-off value using positive and negative serum samples. (a) Interactive dot diagram of cELISA results. The *Y*-axis represents the percentage inhibition (PI) values for 94 negative and 110 positive serum samples. The *X*-axis represents the detection results from the IDEXX commercial kit (1, positive; 0, negative). (b) ROC curve analysis for the cELISA. The area under the curve (AUC) was 0.995 (95% confidence interval [CI]: 0.973–1.000, *p*  < 0.001). The diagnostic sensitivity and specificity of the cELISA were 96.4% and 97.9%, respectively, with an optimal cut-off value of 46.1564% PI.

To establish the cutoff value for the cELISA, a total of 110 clinically confirmed PRV-positive and 94 PRV-negative swine serum samples were analyzed using the cELISA method. The OD_450_ values were recorded, and PI values were subsequently calculated. ROC curve analysis, along with interactive dot diagram analysis, was conducted utilizing MedCalc software (version 20.2) to identify the optimal cutoff value that maximizes both sensitivity and specificity for the developed cELISA. The result indicated that diagnostic sensitivity and specificity of the cELISA were found to be 96.4% and 97.9%, respectively, with an optimal cutoff value set at 46.1564% PI (Figure 6A). The area under the curve (AUC) was determined to be 0.995 (95% confidence interval (CI): 0.973–1.000; *p*  < 0.001; Figure 6B).

### 3.6. Specificity and Sensitivity of the cELISA

**Figure 7:**
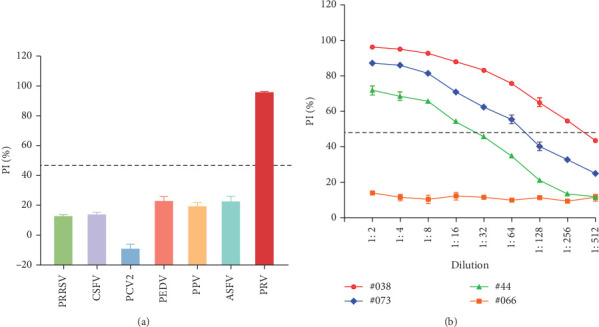
Analysis of the specificity and sensitivity of the cELISA method. (a) Specificity analysis of the mAb 1D11-based cELISA. (b) Sensitivity analysis of the cELISA. The dashed line indicates the established cutoff value of 46.1564% PI.

The specificity of the established method was evaluated by testing serum samples that were positive for six other common swine pathogens (PRRSV, CSFV, PCV2, PEDV, PPV, ASFV) utilizing the optimized cELISA protocol. All serum samples analyzed yielded PI values significantly below the 46.1564% cutoff, indicating that this cELISA method does not cross-react with antibodies against these common porcine pathogens (Figure 7A). This finding confirms the high analytical specificity of this method.

The sensitivity of the assay was evaluated through twofold serial dilutions (1:2 to 1:512) of three PRV-positive serum samples (#038, #073, #44) and one negative serum sample (#066), utilizing the optimized cELISA. Samples #038 and #073 were collected from pigs challenged with PRV HeN1, while sample #44 was obtained from a pig challenged with PRV SC. The cELISA demonstrated high analytical sensitivity, as evidenced by the detection of PRV antibodies in the 1:256 diluted sample #038, the 1:64 diluted sample #073, and the 1:16 diluted sample #44 (Figure 7B).

### 3.7. Agreement With the Commercial ELISA Kit

The diagnostic performance of the cELISA was assessed using a panel of 115 clinical serum samples. These samples were tested with both the commercial IDEXX kit and the cELISA kit. The results demonstrated a high level of agreement (94.78%) between the cELISA kit and the IDEXX kit (Table 2). Furthermore, the kappa value was found to be 0.9, indicating that no significant performance discrepancies were observed between the two kits.

### 3.8. Reproducibility of cELISA

To evaluate the reproducibility of this cELISA, three samples—strong positive, weak positive, and negative—were evaluated using different batches of coated ELISA plates. The intra-assay and inter-assay CVs were calculated. The results indicated that the CV for the PI across intra-assay tests ranged from 0.77% to 1.71%, while the inter-assay CV varied from 1.56% to 7.34%. Both intra- and inter-assay CVs remained below 10%, demonstrating that the gD-cELISA is highly reproducible and reliable for routine diagnostic applications (Table 3).

## 4. Discussion

The global swine industry continues to face significant economic losses from PRV. Effective control and eradication programs rely on rapid, reliable, and high-throughput diagnostic tools. This study successfully addressed this need by developing and validating a novel cELISA for the detection of antibodies against PRV gD. A key factor in the success of this assay was the production of a high-quality recombinant antigen. The use of the HEK293F mammalian expression system was a strategic choice. Unlike prokaryotic systems, mammalian cells perform complex post-translational modifications, such as glycosylation, which are essential for the proper folding and antigenicity of viral envelope proteins. Indeed, PRV gD is known to be a glycoprotein, and expressing it in a system that preserves its native conformation is paramount for developing an assay that accurately detects antibodies raised against the natural virus. The high yield and purity of gD protein provided a solid foundation for all subsequent steps.

The development of a competitive ELISA format offers distinct advantages. It relies on a single, well-characterized mAb, which ensures consistency and reduces the variability associated with polyclonal antisera used in indirect ELISAs. The mAb generated in this study proved to be an ideal reagent, exhibiting high affinity for gD and, crucially, no cross-reactivity with other endemic porcine pathogens. This high degree of specificity is essential for avoiding false-positive results in field settings where co-infections can be common.

The developed gD-cELISA demonstrated excellent diagnostic performance, with a high sensitivity and specificity. These values are highly competitive with existing cELISAs that typically target gE or gB antibodies. gD is a major structural component of PRV and is central to the viral entry process, making it highly immunogenic and a robust target for antibody detection. While gE-based ELISAs are invaluable for DIVA strategies using gE-deleted vaccines, a gD–based assay serves as a powerful complementary tool for confirming infection status, monitoring herd immunity, and conducting large-scale epidemiological surveillance. The high level of agreement (*κ* = 0.90) between the gD–based cELISA and the commercial gB-based IDEXX kit is noteworthy, despite the differing antigen targets. This strong correlation suggests that antibodies against gD and gB are frequently co-produced during PRV infection in swine, likely due to the strong immunogenicity of both glycoproteins. It also indicates that gD-specific antibody responses are a robust marker of PRV exposure, comparable to the well-established gB response. While gB remains a common target in commercial assays, the use of gD provides an independent and complementary diagnostic target. This is particularly valuable in monitoring herds where gB–based vaccines are used, as it offers an alternative serological marker that may help corroborate infection status or vaccine uptake, reinforcing the reliability of PRV surveillance programs.

Despite the promising performance of the gD-cELISA, several limitations should be acknowledged. First, the assay relies on a single mAb (1D11), which may be susceptible to epitope variations in field strains. Although gD is highly conserved, continuous monitoring of its antigenic stability in circulating PRV isolates is warranted. Second, this study focused solely on swine sera; future validation in other susceptible species (e.g., ruminants or carnivores) would be valuable to assess cross-species applicability. Last, while no cross-reactivity was observed with other common swine pathogens, the potential interference from uncharacterized field coinfections cannot be entirely ruled out. These aspects represent important directions for further assay optimization and field deployment.

In conclusion, the gD-cELISA described in this report is a sensitive, specific, and reproducible assay. Its reliance on a stable recombinant antigen and a specific mAb, combined with the simple and rapid ELISA format, makes it highly suitable for routine use in veterinary diagnostic laboratories. This assay represents a valuable addition to the diagnostic arsenal for the control and eventual eradication of PRV. Further validation using a broader range of field samples from different geographical regions is warranted to confirm its utility on a global scale.

## 5. Conclusions

In conclusion, this study successfully developed and validated a novel cELISA (gD-cELISA) for the highly sensitive and specific detection of antibodies against PRV gD. The assay's excellent performance was demonstrated by high sensitivity and specificity and high reproducibility. This gD-cELISA serves as a powerful and practical tool for large-scale serological monitoring, surveillance of herd immunity, and confirmation of infection status, thereby making a valuable contribution to PRV control and eradication programs.

## Figures and Tables

**Table 1:** Optimal reaction conditions for cELISA.

Optimized dilutions and reaction conditions	1D11 mAb–based cELISA
Coating condition	200 ng/well, 4°C 12 h
Blocking condition	2% skim milk, 37°C, 1.5 h
Untested samples	1:2, 37°C, 0.5 h
1D11–HRP	1:200, 37°C, 0.5 h
Chromogenic	100 μL, 37°C, 15 min

**Table 2:** Comparison with commercial ELISA kit.

Samples	cELISA	Number	IDEXX kit		Agreement	Kappa value
			+	−		
Serum	+	62	57	5	94.78%	0.90
	−	53	1	52		

**Table 3:** Intra- and interbatch reproducibility of cELISA based on 1D11 mAb.

Samples	Intra-assay		Interassay	
	Mean ± SD	CV%	Mean ± SD	CV%
Positive 1 (#9)	89.01 ± 0.68	0.77	87.41 ± 1.37	1.56
Positive 2 (#58)	62.88 ± 0.82	1.30	60.25 ± 3.04	5.04
Positive 3 (#143)	45.38 ± 0.77	1.71	46.33 ± 2.08	4.49
Negative 1 (#C)	18.55 ± 0.27	1.46	17.18 ± 1.27	7.34

## Data Availability

The data that support the findings of this study are available from the corresponding author upon reasonable request. The data that support the findings of this study are not publicly available due to restrictions concerning the source of the animal sera.
